# The risk of ventricular catheter misplacement and intracerebral hemorrhage in shunt surgery for hydrocephalus

**DOI:** 10.1186/2045-8118-12-S1-O36

**Published:** 2015-09-18

**Authors:** Linus Hultegård, Asgeir Jakola, Dan Farahmand

**Affiliations:** 1 University of Gothenburg, Sweden; 2 Institute of Neuroscience and Physiology at Sahlgrenska Academy, Sweden

## Introduction

Communicating hydrocephalus is caused by an accumulation of cerebrospinal fluid (CSF) that is often treated with a ventricular shunt. There are few studies on the rates of ventricular catheter (VC) misplacement and postoperative intracerebral hemorrhage (ICH) after shunt surgery. In this study, we evaluated the rate of VC misplacement and ICH after shunt surgery. In addition, VC misplacement was correlated to postoperative ICH and shunt revision within six months, respectively.

## Methods

All patients (n=245) that received a ventriculoperitoneal or ventriculoatrial shunt during January 2012 and December 2014 at the department of neurosurgery at Sahlgrenska University Hospital, Gothenburg, Sweden were included in the study. Patients who did not undergo postoperative imaging (n=14) were excluded. Misplacement was defined as when the tip of the VC was located in the contralateral ventricle or intraparenchymatously. The event of ICH was based on verification of intraparenchymatous blood on an early (<48 h) head CT postoperatively. The shunt revision rate within six months postoperatively was compared between patients with and without misplacement of the VC.

## Results

Misplacement of the VC tip was found in 77 patients (33%); 71 patients with the VC tip in the contralateral ventricle and 6 patients with the VC tip intraparenchymatously. Nine patients (4%) had postoperative ICH verified by imaging of which five (56%) patients had a misplacement of the VC. The revision rate for accurately placed VCs was 17 % compared to 21 % for misplaced VC (n.s.).

## Conclusions

Misplacement of the VC occurred in one third of the shunt insertions; however this did not significantly increase the shunt revision rate. The study showed a fairly high rate of radiologically verified ICH, particularly when the VC was misplaced intraparenchymatously.

